# Current Developments in Dementia Risk Prediction Modelling: An Updated Systematic Review

**DOI:** 10.1371/journal.pone.0136181

**Published:** 2015-09-03

**Authors:** Eugene Y. H. Tang, Stephanie L. Harrison, Linda Errington, Mark F. Gordon, Pieter Jelle Visser, Gerald Novak, Carole Dufouil, Carol Brayne, Louise Robinson, Lenore J. Launer, Blossom C. M. Stephan

**Affiliations:** 1 Institute of Health and Society, Newcastle University Institute of Ageing, Newcastle University, Newcastle upon Tyne, NE2 4AX, United Kingdom; 2 Medical School, Newcastle University, Newcastle upon Tyne, NE2 4HH, United Kingdom; 3 Boehringer Ingelheim Pharmaceuticals, Inc., 900 Ridgebury Road, Ridgefield, Connecticut, 06877, United States of America; 4 Maastricht University, Department of Psychiatry & Neuropsychology, School for Mental Health and Neuroscience, Maastricht, The Netherlands; 5 VU University Medical Centre, Department of Neurology, Alzheimer Centre, Neuroscience Campus, Amsterdam, The Netherlands; 6 Janssen Pharmaceutical Research and Development, 1125 Trenton-Harbourton Road, Titusville, New Jersey, 08560, United States of America; 7 Inserm Research Centre (U897), Team Neuroepidemiology, F-33000, Bordeaux, France; 8 Department of Public Health and Primary Care, Cambridge University, Cambridge, CB2 0SR, United Kingdom; 9 Laboratory of Epidemiology, Demography and Biometry, National Institute on Aging, National Institutes of Health (NIH), Bethesda, Maryland, United States of America; "Mario Negri" Institute for Pharmacological Research, ITALY

## Abstract

**Background:**

Accurate identification of individuals at high risk of dementia influences clinical care, inclusion criteria for clinical trials and development of preventative strategies. Numerous models have been developed for predicting dementia. To evaluate these models we undertook a systematic review in 2010 and updated this in 2014 due to the increase in research published in this area. Here we include a critique of the variables selected for inclusion and an assessment of model prognostic performance.

**Methods:**

Our previous systematic review was updated with a search from January 2009 to March 2014 in electronic databases (MEDLINE, Embase, Scopus, Web of Science). Articles examining risk of dementia in non-demented individuals and including measures of sensitivity, specificity or the area under the curve (AUC) or c-statistic were included.

**Findings:**

In total, 1,234 articles were identified from the search; 21 articles met inclusion criteria. New developments in dementia risk prediction include the testing of non-APOE genes, use of non-traditional dementia risk factors, incorporation of diet, physical function and ethnicity, and model development in specific subgroups of the population including individuals with diabetes and those with different educational levels. Four models have been externally validated. Three studies considered time or cost implications of computing the model.

**Interpretation:**

There is no one model that is recommended for dementia risk prediction in population-based settings. Further, it is unlikely that one model will fit all. Consideration of the optimal features of new models should focus on methodology (setting/sample, model development and testing in a replication cohort) and the acceptability and cost of attaining the risk variables included in the prediction score. Further work is required to validate existing models or develop new ones in different populations as well as determine the ethical implications of dementia risk prediction, before applying the particular models in population or clinical settings.

## Introduction

Dementia is a complex disease often caused by a combination of genetic and environmental risk factors. Although many risk factors for the occurrence and progression of dementia have been identified, their utility for determining individual risk through dementia prediction models remains unclear.

Numerous models for predicting dementia, and more specifically Alzheimer’s Disease (AD), have been developed[1]. Such models could be used to refine inclusion criteria for clinical trials, focus treatment and intervention more effectively and help with health surveillance. A systematic review published in 2010 identified over 50 different dementia risk prediction models[1]. The models differed in the number and type of variables used for risk score calculation, follow-up time, disease outcome and model predictive accuracy. The review concluded that no model could be recommended for dementia risk prediction largely due to methodological weaknesses of the published studies. Model development had generally been based on small cohorts, restricted to Caucasians, and at the time of the review there had been a lack of objective and unbiased model evaluation, such as external validation.

Over the last five years, research into dementia risk prediction has greatly expanded and dementia prevention is a high policy priority in many countries. In order for clinicians, researchers and policy makers to keep up to date on relevant findings and make decisions about which model to apply to identify those high risk of future dementia, it is necessary to have an accurate knowledge of model development (including component variables and validation work), discriminative accuracy, and sensitivity and specificity of cut-off scores. In this review, based on the results of an updated literature search, we aim to evaluate the latest developments in dementia risk prediction modelling including a critique of the variables selected for model inclusion and an assessment of model prognostic performance.

## Methods

### Search Strategy

This review has been undertaken with adherence to the PRISMA statement[2]. MEDLINE, Embase, Scopus and ISI Web of Science were searched using combinations of the following terms and mapped to Medical Subject Headings (MeSH): “dementia”, “Alzheimer disease”, “Alzheimer and disease”, “predict”, “develop”, “incident”, “sensitivity”, “specificity”, “ROC” and “area under the curve”. The search included all literature published between the 1 January 2009 to 17 March 2014 (see Table 1 for the search strategy). Only articles published in English were considered. Additional articles were identified from the reference lists of eligible studies and relevant reviews. All articles published from January 2009 –November 2009 were also removed as this was covered in the original review.

**Table 1:** Search Strategy.

1. exp dementia
2. predict$.mp.
3. develop$.mp.
4. inciden$.mp.
5. sensitivity.mp.
6. specificity.mp.
7. "sensitivity and specificity"
8. 2 or 3 or 4 or 5 or 6 or 7
9. receiver operating characteristic
10. ROC.mp.
11. area under the curve
12. AUC.mp.
13. concordance statistic.mp.
14. c statistic.mp.
15. 9 or 10 or 11 or 12 or 13 or 14
16. 1 and 8 and 15
17. limit 16 to yr = "2009-Current"

### Selection of Studies

Selection of articles followed the same protocol as the 2010 review[1]. Two authors (ET and SH) independently searched publications using the following inclusion criteria: the sample was population-based and the article examined risk of dementia in non-demented individuals and included measurements of sensitivity, specificity or discrimination (i.e., area under the curve: AUC or c-statistic). Cross-sectional, case-control and clinical-based studies were excluded, as were studies that restricted the baseline sample by cognitive criteria other than dementia status; for example studies were excluded that focused only on subjects with Mild Cognitive Impairment (MCI). Articles where the outcome was a combined cognitive group, for example dementia cases combined with individuals with MCI, were also excluded. Titles and abstracts were searched first, followed by the full text of any identified articles. Where duplicate studies were identified, details of all novel risk models and their performance indices (AUC/c-statistic, sensitivity or specificity) were extracted from each publication and reported separately. One set of duplicate publications was found[3, 4]. However, the first paper[3] only presented the model and did not undertake testing. This paper was therefore excluded. Disagreements were solved by consensus between the two authors (ET and SH), or by a third author (BCMS) if the disagreement could not be resolved.

### Data Extraction

Two authors (ET and SH) independently extracted information from each study including: sample, country, length of follow-up, baseline age, sex distribution, outcome tested (e.g., all-cause dementia vs. dementia subtypes), the components of each prediction model, discriminative accuracy (AUC or c-statistic), and where available, sensitivity and specificity estimates of cut-off scores and positive/negative likelihood ratio (LR+ or LR-, respectively). Two authors (ET, SH) independently assessed the quality of the included studies using an adapted version of the Newcastle-Ottawa Scale (NOS) for non-randomized studies, specifically cohort studies[5], as endorsed by the Cochrane collaboration [6]. The NOS uses a star rating system to assess selection, comparability and outcome criteria. Items describing a non-intervention cohort were excluded and therefore the total ranking was out of 6 (rather than 9).

## Results

### Included Studies

**Fig 1:**
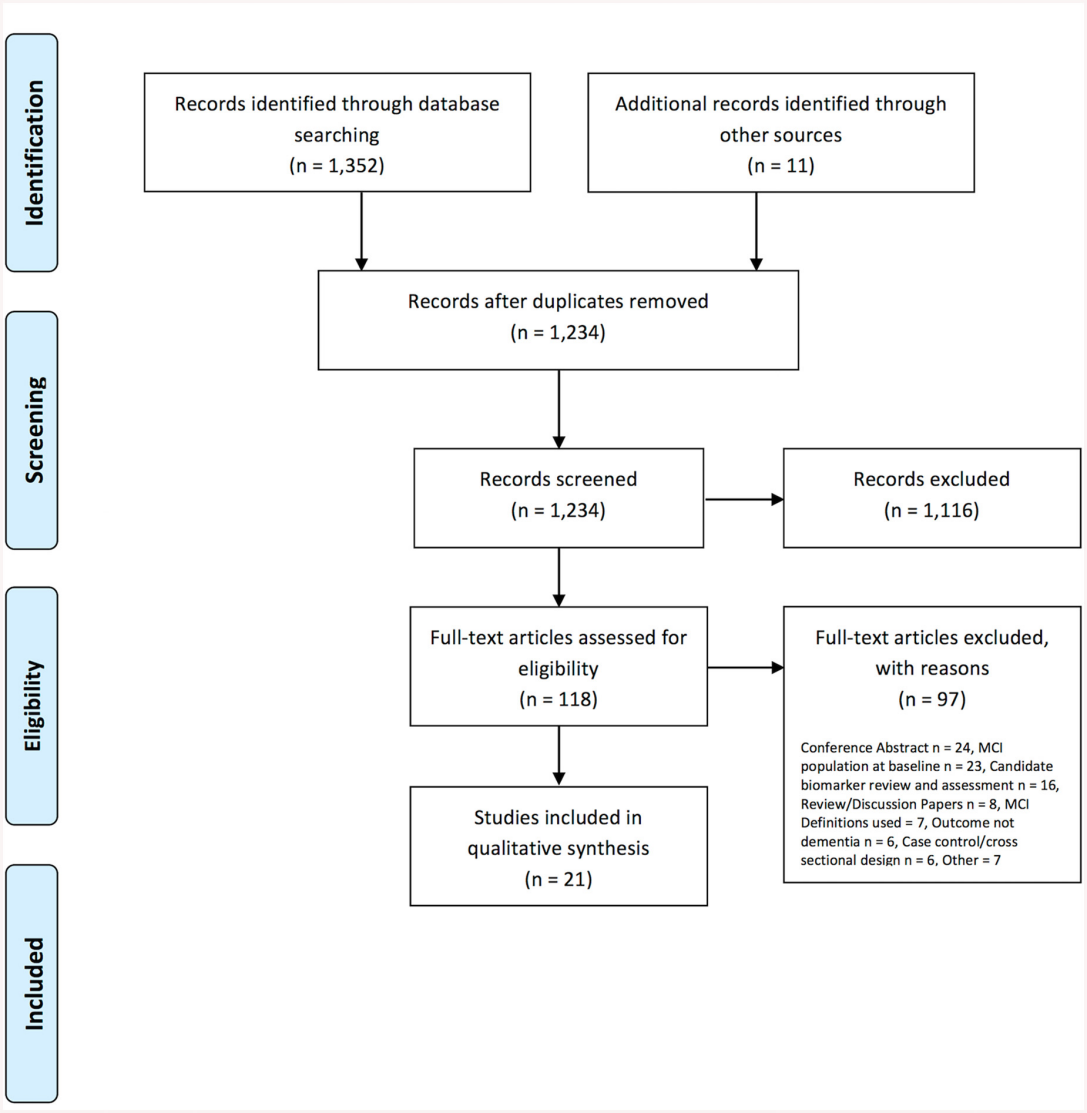
PRISMA (2009) flow diagram of article selection.

A total of 1,234 articles were identified from the electronic literature search (after removing duplicate publications) and 11 articles were identified from other sources. In total, 21 articles describing dementia risk prediction models were included in this review (Fig 1).

Table 2 summarises the study characteristics and predictive models. 13 new study populations[7–20] have been used to create risk models compared to the first review (all literature till 2009)[1]. There was however some overlap including use of data from the Cardiovascular Health Cognition Study[20, 21], Leipzig Longitudinal Study of the Aged (LEILA 75+)[22], Einstein Aging Study[23], Vienna TransDanube Aging[24], Canadian Study of Health and Aging[25] and the Kungsholmen Project[4]. Sample sizes ranged from 194[11] to 29,961[13]. Follow-up duration ranged from 1.4[22] to 20 years[7]. Follow-up rate ranged from 66%[12] to 86%[19], with 10 studies[4, 7, 11, 14, 15, 17, 20, 21, 23, 25] not reporting attrition or dropout. In ten studies[7, 10, 12–14, 16, 20–22, 26] the outcome tested was all-cause dementia, in nine studies[8, 9, 11, 15, 17–19, 23, 24] the outcome was AD and two studies had separate models for both AD and all-cause dementia [4, 25]. The number of predictors ranged from one[22] to 19[25].

**Table 2:** Characteristics of included studies and reported dementia risk prediction models.

Sample	Follow- up sample	Number of events (type)	Diagnostic criteria	Dementia subtypes	Follow-up (years) and follow-up rate	Baseline age (years)	Model	Sn (%)	Sp (%)	AUC	Validation	Cal	Ex val	Follow-up rate	Dementia subtypes tested	Additio-nal notes	Quality Assessment: Newcastle Ottawa Scale
	**(1) Demographic and Cognitive Models**																
Paquid (French Cohort Study) (7)	2882	804	DSM-III-R	No	20 years (3 and 10 year models shown)	>65	LEL + (age, ≥75 years, 4-IADLs, episodic memory subtest) at 3 years	85.0	39.0	0.75	Yes, bootstrap case cross validation with bias reduction	No	No	No statement	No	Sn and Sp for a cut point corresp-onding to Sn = 0.85 in the learning sample.	*****
							LEL + (age, ≥75 years, 4-IADLs score, DSST) at 10 years	82.0	50.0	0.75	Yes (as above)	No	No	No	No		
							HEL + (age, ≥75 years, memory complaints (new simple information), IST, DSST, episodic memory subtest) at 3 years	83.0	69.0	0.85	Yes (as above)	No	No	No	No		
							HEL + (age, ≥75 years, IST, BVRT, DSST, episodic memory subtest) at 10 years	84.0	52.0	0.78	Yes (as above)	No	No	No	No		
The Canadian Study of Health and Ageing(26)	284	75	DSM-III-R	Probable AD 31, possible AD 20, vascular dementia 18, FTD 2, dementia NOS 3	10 years	≥65	Age, education, gender, RAVLT, short delay recall, WAIS-R digit symbol: Cut off 0.19	84.0	64.0		Yes, bootstrap-ping 1000 samples	No	No	19% lost at follow-up, 6% refused, 55% died at follow-up	No	0.23 cut-off optimal for 10 years; 0.22 optimal for 5 years. Using the bootstrap method, for the 10year study, the potential overestimation was 0.0211 giving validated ROC performance estimates of 0.785–0.0211 = 0.764. For the 5year study, the potential overestimation was 0.0136 producing a performance estimate of 0.807. The small sizes of the overestimations confirm the stability of the predictive models	******
							Age, education, gender, RAVLT, short delay recall, WAIS-R digit symbol: Cut off 0.21	80.0	68.0		Yes, bootstrap-ping 1000 samples	No	No				
							Age, education, gender, RAVLT, short delay recall, WAIS-R digit symbol: Cut off 0.23	78.0	72.0	0.79	Yes, bootstrap-ping 1000 samples	No	No				
							Age, education, gender, RAVLT, short delay recall, WAIS-R digit symbol: Cut off 0.25	71.0	74.0		Yes, bootstrap-ping 1000 samples	No	No				
							Age, education, gender, RAVLT, short delay recall, WAIS-R digit symbol: Cut off 0.27	64.0	77.0		Yes, bootstrap-ping 1000 samples	No	No				
	634	148	DSM-III-R	Probable AD 60, possible AD 36, VaD 40, PD 4, FTD 1, dementia due to HI 1, dementia NOS 6	5 years	≥65	Age, education, gender, WMS information, RAVLT, short delay recall, Animal fluency, WAIS-R digit symbol: Cut off 0.17	79.0	70.0		Yes, bootstrap-ping 1000 samples	No	No				
							Age, education, gender, WMS information, RAVLT, short delay recall, Animal fluency, WAIS-R digit symbol: Cut off 0.19	76.0	71.0		Yes, bootstrap-ping 1000 samples	No	No				
							Age, education, gender, WMS information, RAVLT, short delay recall, Animal fluency, WAIS-R digit symbol: Cut off 0.22	75.0	74.0	0.82	Yes, bootstrap-ping 1000 samples	No	No				
							Age, education, gender, WMS information, RAVLT, short delay recall, Animal fluency, WAIS-R digit symbol: Cut off 0.23	70.0	78.0		Yes, bootstrap-ping 1000 samples	No	No				
							Age, education, gender, WMS information, RAVLT, short delay recall, Animal fluency, WAIS-R digit symbol: Cut off 0.25	65.0	80.0								
Community dwelling, Massach-usetts(8)	342	Not stated	Consensus Diagnosis (dementia) and classified by NINCDS-ARDA and NINDS-AIREN	AD	Mean 7.4 years	≥65	CDR-sum-of-boxes (CDR-SB) adjusted for age, gender and education Clinician based			0.78	No	No	No	Follow-up >80%	No	None	******
							CDR-sum-of-boxes (CDR-SB) adjusted for age, gender and education Algorithm based			0.76							
	**(2) Cognitive Models (Einstein Aging Study Also Contains Multivariable Models)**																
German Study on Aging Cognition and Dementia in Primary Care patients (AgeCoDe)(9)	1504	70	DSM-IV (clinical diagnosis)	50 AD, 14 VaD, 6 NOS	18 months	≥75	CERAD Word-List Learning	87.8	72.4	0.84	No	No	No	Follow-up rate of 60%	No	None	*****
							CERAD Word-List Recall	85.7	62.3	0.84	No	No	No				
							CERAD Total Score	85.7	83.1	0.89	No	No	No				
							CERAD Total Score 2 (including Constructional Praxis Recall	89.8	78.8	0.89	No	No	No				
							CERAD Forward-Selection Mode	88.0	81.6	0.88	No	No	No				
							MMSE	87.8	72.1	0.82	No	No	No				
Einstein Aging Study (EAS) (all individuals had memory complaints) (23)	854	86 (AD)	DSM-IV (clinical diagnosis)	AD	2–4 years (mean 4.2)	≥70 (mean 78.8)	FCSRT-FR 2 years			0.87	Yes, all models bootstrapped	No	No	No statement	No	Cut scores are Youden’s index cut. (FCSRT-FR 28); Cut scores are Youden’s index cut. (FCSRT-FR 28) LM-IR 16)	*****
							Cut off score 32	94.1	47.0			No	No				
							Cut off score 31	91.3	55.7			No	No				
							Cut off score 30	89.7	60.8			No	No				
							Cut off score 29	88.3	68.6			No	No				
							Cut off score 28	85.7	73.6			No	No				
							Cut off score 27	80.8	78.4			No	No				
							Cut off score 26	73.1	82.6			No	No				
							Cut off score 25	67.6	85.5			No	No				
							Cut off score 24	63.3	88.5			No	No				
							Cut off score 23	59.1	91.3			No	No				
							Cut off score 22	53.7	94.2			No	No				
							FCSRT-FR 3 years			0.88		No	No				
							Cut score 32	96.2	48.2			No	No				
							Cut score 31	94.3	57.1			No	No				
							Cut score 30	92.0	52.2			No	No				
							Cut score 29	87.6	70.0			No	No				
							Cut score 28	82.1	75.1			No	No				
							Cut score 27	76.4	79.6			No	No				
							Cut score 26	67.9	83.6			No	No				
							Cut score 25	62.8	86.5			No	No				
							Cut score 24	58.2	89.4			No	No				
							Cut score 23	54.6	92.3			No	No				
							Cut score 22	49.7	95.1			No	No				
							FCSRT-FR 4 years			0.89		No	No				
							Cut score 32	97.2	49.6			No	No				
							Cut score 31	95.9	58.8			No	No				
							Cut score 30	93.8	64.0			No	No				
							Cut score 29	89.6	71.8			No	No				
							Cut score 28	84.9	77.0			No	No				
							Cut score 27	80.9	81.7			No	No				
							Cut score 26	73.2	85.7			No	No				
							Cut score 25	66.7	88.3			No	No				
							Cut score 24	59.5	90.9			No	No				
							Cut score 23	52.5	93.4			No	No				
							Cut score 22	44.1	95.8			No	No				
							LM-IR 2 years			0.78		No	No				
							Cut score 22	94.4	39.9			No	No				
							Cut score 21	92.4	46.0			No	No				
							Cut score 20	89.9	50.7			No	No				
							Cut score 19	83.5	57.0			No	No				
							Cut score 18	79.2	61.5			No	No				
							Cut score 17	74.6	66.9			No	No				
							Cut score 16	71.9	71.1			No	No				
							Cut score 15	66.7	75.1			No	No				
							Cut score 14	57.5	80.7			No	No				
							Cut score 13	49.5	84.0			No	No				
							Cut score 12	43.2	86.9			No	No				
							Cut score 11	35.1	89.5			No	No				
							LM-IR 3 years			0.77		No	No				
							Cut score 22	92.9	40.7			No	No				
							Cut score 21	90.8	46.9			No	No				
							Cut score 20	88.6	51.7			No	No				
							Cut score 19	84.4	58.1			No	No				
							Cut score 18	80.8	62.6			No	No				
							Cut score 17	76.7	68.1			No	No				
							Cut score 16	72.3	72.2			No	No				
							Cut score 15	65.1	76.1			No	No				
							Cut score 14	52.2	81.3			No	No				
							Cut score 13	43.9	84.5			No	No				
							Cut score 12	38.9	87.4			No	No				
							Cut score 11	32.9	89.9			No	No				
							LM-IR 4 years			0.75		No	No				
							Cut score 22	88.2	41.1			No	No				
							Cut score 21	83.7	47.2			No	No				
							Cut score 20	80.4	51.9			No	No				
							Cut score 19	76.3	58.5			No	No				
							Cut score 18	72.9	63.0			No	No				
							Cut score 17	69.8	68.7			No	No				
							Cut score 16	66.7	72.9			No	No				
							Cut score 15	61.4	76.8			No	No				
							Cut score 14	50.8	82.1			No	No				
							Cut score 13	44.1	85.3			No	No				
							Cut score 12	38.8	88.1			No	No				
							Cut score 11	33.0	90.6			No	No				
							FCSRT-FR and LM-IR of the Wechsler Memory Scale-revised. (3 years) Model 1: FCSRT-FR adjusted for age, sex, education, race			0.85		No	No				
							Model 2: Model 1 + LM-IR. Among the subgroup with APOE e4 status available			0.86		No	No				
							Model 3: FCSRT-FR adjusted for age, sex, education, race;			0.86		No	No				
							Model 4: Model 3 + LM-IR			0.87		No	No				
							Model 5: Model 4 + APOE e4 status			0.87		No	No				
Leipzig Longitudinal Study of the Aged (LEILA 75+)(22)	384	28 (dementia)	DSM-IV (clinical diagnosis)	None	Average 1.4 years followed up for 5 assessments, followed-up at 3rd assessment	≥75, dementia cases (mean 85.16), dementia free-cases (mean 83.03)	CDT			0.70	No	No	No	Follow-up rate 70%	No	None	*****
							CDT = 4	21.0	97.0		No	No	No		No		
							CDT ≥3	68.0	65.0		No	No	No		No		
							CDT = 2	75.0	57.0		No	No	No		No		
							MCI			0.77	No	No	No		No		
The Leukoar-aiosis and Disability (LADIS) Study(10)	480	90 (dementia)	DSM-IV	No	3 years	65–84, demen-tia cases (mean 75.9), no demented (mean 73.7)	**Batteries**							75% follow-up rate		None	*****
							MMSE	29.0	87.0	0.79	No	No	No		No		
							ADAS-Cog	20.0	84.0	0.79	No	No	No		No		
							VADAS	25.0	88.0	0.82	No	No	No		No		
							VADAS extension	8.0	82.0	0.79	No	No	No		No		
							**Single Tests**				No	No	No		No		
							Trail-making (Part B-A)		86.0	0.70	No	No	No		No		
							Stroop (part 3–2)	62.0	86.0	0.69	No	No	No		No		
							Word immediate recall	71.0	85.0	0.68	No	No	No		No		
							Delayed recall	20.0	85.0	0.71	No	No	No		No		
							Word recognition	54.0	85.0	0.69	No	No	No		No		
							Constructional Praxis		85.0	0.62	No	No	No		No		
							Ideational praxis	40.0	85.0	0.59	No	No	No		No		
							Naming		84.0	0.56	No	No	No		No		
							Orientation	65.0	84.0	0.70	No	No	No		No		
							Symbol digit	68.0	87.0	0.78	No	No	No		No		
							Digit Span	50.0	85.0	0.64	No	No	No		No		
							Digit cancellation	55.0	86.0	0.73	No	No	No		No		
							Maze	58.0	86.0	0.71	No	No	No		No		
							Verbal Fluency	45.0	86.0	0.75	No	No	No		No		
African American and Caucasian Primary Care Patients (Geriatric Ambulatory Practice)(11)	194	28 (dementia)	DSM-IV (clinical diagnosis)	AD and non-AD dementia. 11 probable AD, 12 possible AD, 9 mixed (AD and VaD), 10 with VaD, 6 with other dementias, 2 insuffi-cient evidence for subtyping	Median 2.6 years	Mean 78.3	FCSRT-FR			0.81	No	No	No	No statement	No	Non-AD (n = 16) Total Recall = 45.0 AD (n = 32) Total recall; Non-AD Free recall 21.5. AD free recall = 17.5 (no difference between non-AD and AD in free recall p = 0.063	*****
							FCSRT-TR			0.65	No	No	No		No		
Medical Research Council Cognitive Function and Ageing Study (MRC CFAS)(12)	1347	137 (dementia)	AGECAT organicity rating case level of ≥3 (equivalent to 0.72DSM-III-R)	No	2 years	No deme-ntia 74.7, demen-tia 80.5	Memory (remote range 0–6)	81.0	66.0	0.74	No	No	No	66% follow-up rate	No	None	*****
							Memory (recent range 0–4)	73.0	71.0	0.72	No	No	No		No		
							Memory (learning range 0–17)	63.0	78.0	0.71	No	No	No		No		
							Orientation (range 0–10)	91.0	40.0	0.65	No	No	No		No		
							Language comprehension (range 0–9)	96.0	16.0	0.63	No	No	No		No		
							Language expression (range 0–21)	87.0	42.0	0.65	No	No	No		No		
							Attention and calculation (range 0–8)	71.0	58.0	0.67	No	No	No		No		
							Praxis (range 0–8)	90.0	34.0	0.68	No	No	No		No		
							Abstraction (range 0–8)	64.0	62.0	0.63	No	No	No		No		
							Perception (range 0–8)	78.0	53.0	0.65	No	No	No		No		
							Composite score (CAMCOG total range 0–103)	72.0	80.0	0.76	No	No	No		No		
							Composite score (Memory 0–27)	76.0	77.0	0.77	No	No	No		No		
							Composite score (non-memory range 0–76)	65.0	79.0	0.72	No	No	No		No		
	**(3) Multivariable Models**																
Type 2 diabetes patients from the Kaiser Permanente Northern California (KPNC) Diabetes Registry, Oakland, CA, USA(13)	29 961	5173 (dementia)	ICD-9 codes for dementia	No	10 years	≥60, demen-tia cases (mean 74.2), demen-tia free cases (mean 69.7)	Age, education, mircovascular disease, diabetic foot, cerebrovascular disease, cardiovascular disease, acute metabolic event and depression			0.74	No	Yes, Hosmer-Lemeshow χ² = 15.1	Yes	48% died, 24% had been diagnosed with dementia	No	External validation: Cohort of 2413 patients (aged .60 years) with type 2 diabetes who are members of the Pathways study cohort from Washington, USA. AUC for validation cohort 0.75	******
German Study on Aging Cognition and Dementia in Primary Care patients (AgeCoDe)(19)	3055	193 (AD)	DSM-IV (clinical diagnosis) for dementia; NINCDS-ADRDA for AD	AD	3 follow-ups at 18 month intervals Mean follow-up 3.81	≥75, mean 80.1	Full model: age, sex, SMI, verbal fluency, delayed recall, MMSE, IADL.	85.5	63.8	0.84	Yes	No	No	86.2% follow-up rate	No	AUC for validation cohort 0.79 (non-significant compared to first cohort).	******
							Full model: age, sex, SMI, verbal fluency, delayed recall, MMSE, IADL, +ApoE4 status			0.85							
Vienna TransDa-nube Aging (VITA) Study (24)	296	65 (possible or probable AD)	NINCDS-ADRDA for AD	AD	60 months	75, no AD mean 75.7, AD mean 75.7	A1 (depressed mood)			0.51	No	No	No	67% follow-up rate	No	In logistic regression only A2 significant, but only 12 patients had a positive value for loss of interest	*****
							A2 (loss of interest)	10.4	97.8	0.54	No	No	No		No		
							A3 (change of appetite)			0.50	No	No	No		No		
							A4 (sleep disturbance)			0.53	No	No	No		No		
							A5 (psychomotor change)			0.52	No	No	No		No		
							A6 (loss of energy)			0.51	No	No	No		No		
							A7 (worthlessness)			0.50	No	No	No		No		
							A8 (concentration difficulty)			0.50	No	No	No		No		
							MODEL: A2 (loss of interest) + APOE e4 + folic acid + education			0.63	No	No	No		No		
The Hisayama Study, Japan (15)	523	136	DSM-III-R	Mixed 8, AD 81, VaD 39	17 years	Mean 66.8	MODEL: Age + sex + education + smoking + alcohol intake + systolic blood pressure + use of antihypertensive agents + HbA1c + serum total cholesterol + BMI and regular exercise			0.68	No	No	No	No statement	No	Net reclassification improvement: 0.18, (P0.01); integrated discrimination improvement: 6.25, P<0.001) also confirmed this improvement in AD risk assessment.	*****
							MODEL: Age + sex + education + smoking + alcohol intake + systolic blood pressure + use of antihypertensive agents + HbA1c + serum total cholesterol + BMI and regular exercise + APOE			0.74	No	No	No		No		
Medicare Recipients 65+ in Northern Manhattan, New York(16)	1051	92 possible and probable LOAD, 80 probably LOAD	DSM-IV. Possible LOAD: when most likely LOAD but could be other disorders. Probable LOAD: dementia could not be explained by another disorder	LOAD	Mean follow-up 4.2 years at 18 month intervals	75.66 without LOAD, 79.74 with LOAD	Demographic (Age, sex, education, ethnicity) + Health (Diabetes, hypertension, HDL-C, waist-to-hip ratio) + Lifestyle (Smoking)			0.79	No	No	No	>80% follow-up rate	No	None	******
							Demographics			0.72							
							Demographics + APOE			0.75							
Rotterdam Study(17)	5700	462	DSM-III or DSM-IV for dementia and NINCDS-ADRDA for AD	AD	Mean 9.3 years	69	Age, sex			0.83	No	No	No	No statement	No	None	*****
							Age, sex, APOE e4			0.85	No	No	No		No		
							Age, sex, APOE e4, PICALM, CLU			0.85	No	No	No		No		
Cardiovas-cular Health Study(17)	2429	435		AD	Mean 6.8 years	75	Age, sex			0.67	No	No	No		No		
							Age, sex, APOE e4			0.70	No	No	No		No		
							Age, sex, APOE e4, PICALM, CLU			0.71	No	No	No		No		
Rotterdam Study(18)	5507	359 (AD)	DSM-III-R for dementia; NINCDS-ADRDA for AD	AD	10 years	45–99	Model 1 (age and sex)			0.79	No	No	No	>80% follow-up rate	No	None	******
							Model II (age, sex and APOE e4 carriership)			0.81	No	No	No		No		
							Model III (age, sex, APOE e4 carriership, genetic risk* score without APOE)			0.82	No	No	No		No		
Cardiovascular Health Cognition Study(21)	3252	451	Review committee of neurologists and psychiatrists (clinical)	None	6 years	Not specified	Age (75–79 and 80–100), Delayed recall <2 of 3 words, incorrectly copying intersecting pentagons, incorrectly taking or folding paper, inability to name 10 four legged animals in 30s, self-reported "trouble keeping mind on things" > = 3 d/wk, stroke, peripheral arterial disease, CABG, BMI <18.5, lack of current alcohol consumption, constant			0.77	No	No	No	No statement	No	None	****
							Age (continuous variable), Delayed recall <2 of 3 words, incorrectly copying intersecting pentagons, incorrectly taking or folding paper, inability to name 10 four legged animals in 30s, self-reported "trouble keeping mind on things" > = 3 d/wk, stroke, peripheral arterial disease, CABG, BMI <18.5, lack of current alcohol consumption, constant			0.78	No	No	No		No		
							Age 75–79, Age 80–100, Delayed recall <2 of 3 words, incorrectly copying intersecting pentagons, incorrectly taking or folding paper, inability to name 10 four legged animals in 30s, self-reported "trouble keeping mind on things" > = 3 d/wk, stroke, peripheral arterial disease, CABG, BMK <18.5, lack of current alcohol consumption, walking speed, constant			0.78	No	No	No		No		
Kaiser Permanente Medical Care Program of Northern California (KPNC) (14)	9480	2767	ICD-9	None	36.9 years	40–55 (mean 46.1)	CAIDE score (age, education, sex, cholesterol, BMI, systolic blood pressure)				Yes	Yes, Hosmer-Lemeshow	Yes	No statement	None	Note: this paper is an external validation of the original CAIDE score; No improvement of NRI or IDI with additional variables, similar to the AUC	*****
							Logistic Analysis										
							CAIDE			0.75							
							CAIDE (Asian)			0.81							
							CAIDE (Black)			0.75							
							CAIDE (White)			0.74							
							CAIDE + central obesity			0.75							
							CAIDE + depressed mood			0.75							
							CAIDE + diabetes mellitus			0.75							
							CAIDE + head trauma			0.75							
							CAIDE + poor lung function			0.75							
							CAIDE + smoking			0.75							
							Cox Analysis										
							CAIDE			0.67							
							CAIDE + central obesity			0.67							
							CAIDE + depressed mood			0.67							
							CAIDE + diabetes mellitus			0.67							
							CAIDE + head trauma			0.67							
							CAIDE + poor lung function			0.67							
							CAIDE + smoking			0.67							
Cardiovascular Health Study (CHS), Framingham Heart Study (FHS), the Health and Retirement Study (HRS), Sacramento Area Latino Study on Aging (SALSA)(20)	CHS 2794, FHS 2411, HRS 13889, SALSA 1125	Not stated	Cognitive impairment in at least two domains that reflected a decline from prior levels and sufficient severity to affect daily function (CHS, FHS and SALSA—Consensus Committee Review, HRS, validated cut points on a brief cognitive battery)	No	6 years	71–73	Age, education, history of stroke, diabetes mellitus, BMI, assistance needed with money or medications, depressive symptoms				Yes	Yes	No	No statement	No	Predictive factors identified in each study predictive of dementia. Those that were consistent between cohorts used in final model.	*****
							CHS Cohort			0.68							
							CHS (whites)			0.70							
							CHS (blacks)			0.65							
							FHS cohort			0.77							
							HRS cohort			0.76							
							HRS (whites)			0.75							
							HRS (blacks)			0.70							
							HRS (Latino)			0.71							
							SALSA cohort			0.78							
Cardiovascular Health Study (CHS), Framingham Heart Study (FHS), the Health and Retirement Study (HRS), Sacramento Area Latino Study on Aging (SALSA)(20)	CHS 2794, FHS 2411, HRS 13889, SALSA 1125	Not stated	Cognitive impairment in at least two domains that reflected a decline from prior levels and sufficient severity to affect daily function (CHS, FHS and SALSA—Consensus Committee Review, HRS, validated cut points on a brief cognitive battery)	No	6 years	71–73	ANU-ADRI (age, gender, education, diabetes, traumatic brain injury, cognitive activity, social network and engagement, smoking, alcohol, physical activity, fish intake, depression symptoms)				Yes	No	No	No statement	AD for CAIDE	3 separate validation cohorts used for original ANU-ADRI Risk Index Ex val of CAIDE index	*****
							MAP (ANU-ADRI minus fish intake and depressive symptoms)			0.73							
							KP (ANU-ADRI minus cognitive activity, physical activity, fish intake and depressive symptoms).			0.64							
							CVHS (ANU-ADRI minus traumatic brain injury, cognitive activity, social network and engagement)			0.74							
							Common variables (age, sex, education, diabetes, smoking and alcohol)										
							MAP			0.73							
							KP			0.67							
							CVHS			0.69							
							CAIDE (AD)										
							MAP			0.49							
							KP			0.53							
							CVHS			0.57							
							CAIDE (Dementia NOS)										
							MAP			0.49							
							KP			0.54							
							CVHS			0.57							
							CAIDE (minus BMI) (AD)										
							MAP			0.54							
							KP			0.53							
							CVHS			0.58							
							CAIDE (minus BMI) (Dementia NOS)										
							MAP			0.54							
							KP			0.54							
							CVHS			0.59							
							CAIDE (minus BMI and cholesterol) (AD)										
							MAP			0.55							
							KP										
							CVHS			0.58							
							CAIDE (minus BMI and cholesterol) (Dementia NOS)										
							MAP			0.55							
							KP										
							CVHS			0.60							
Canadian Study of Health and Aging (CSHA)(25)	7239	194 AD at 5 years 106 dementia NOS, 222 at 10 years 85 dementia NOS	NINCDS-ADRA, DSM-III-R	AD	5 and 10 years	≥65	Frailty Index, Non-traditional Risk Factors Index (FI-NTRF) (19 variables of health measures (deficits))								AD	None	****
							5 years AD			0.64	No	No	No	Not stated			
							10 years AD			0.66	No	No	No	Not stated			
							5 years dementia NOS			0.64	No	No	No	Not stated			
							10 years dementia NOS			0.66	No	No	No	Not stated			

* Genetic Risk Score includes the following: CLU, PICALM, BIN1, CR1, ABCA7, MS4A6A, MS4A4E, CD2AP, EPHA1, and CD33.

**Abbreviations:** 4-IADL, Four instrumental activities of daily living; AD, Alzheimer disease; ADAS, Alzheimer's Disease Assessment Scale; ADAS-Cog, Alzheimer's Disease Assessment Scale—cognitive subscale; ANU-ADRi, Australian National University AD Risk Index; APOE, apolipoprotein E; AUC, Area Under the Receiver Operating Characteristic Curve; BVRT, Benton Visual Retention Test; BMI, Body Mass Index; CAIDE, Cardiovascular Risk Factors, Aging and Dementia Study; Cal, calibration; CAMCOG Cambridge Cognitive Examination; CDT, Clock Drawing Test; CERAD, Consortium to Establish a Registry for ADDSM, Diagnostic and Statistical Manual of Mental Disorders; DSST, Digit Symbol Substitution Test; Ex val, external validation; FCSRT-FR, Free recall score from the Free and Cued Selective Reminding Test; FCSRT-TR, Total recall score from the Free and Cued Selective Reminding Test; FTD, Frontotemporal Dementia; GDS, Geriatric Depression Scale; HEL, High Education Level; HI, Head Injury; IAD, Instrumental Activities of Daily Living Scale; ICD-9, International Classification of Diseases, Ninth Revision; IST, Isaacs Set Test; IDSR, Intra-categorical Delayed Selective Reminding test; LEL, Low educational Level, LOAD, Late-onset AD; LM-IR, Logical Memory I immediate recall; MCI, Mild Cognitive Impairment; MMSE, Mini Mental State Examination; NINCDS–ADRDA, Alzheimer's Criteria National Institute of Neurological and Communicative Disorders and Stroke and the Alzheimer's Disease and Related Disorders Association; NINCDS–AIREN, Vascular Dementia Criteria National Institute of Neurological Disorders and Stroke and Association Internationale pour la Recherché et l'Enseignement en Neurosciences; NOS, not otherwise specified; RAVLT, Rey Auditory–Verbal Learning Test; SMI, Subjective Memory Impairment; Sn, Sensitivity; Sp, Specificity; VaD, Vascular Dementia; VADAS, Vascular Dementia Assessment Scale; WAIS-R, The Wechsler Adult Intelligence Scale-Revised; WMS, Wechsler Memory Scale.

### Quality Assessment

Articles were assessed on selection, comparability and outcome (out of a maximum of six stars). In total, six[8, 13, 16, 18, 19, 26] articles scored six stars (maximum), 13 scored five stars[4, 7, 9–12, 14, 15, 17, 20, 22–24] and two[21, 25] scored four stars. This indicates that most articles were of high or moderate quality. Star ratings for each article are shown in Table 2.

### Model Development

Most models have been derived using Logistic Regression[7, 9, 10, 21, 24–26] or Cox Proportional Hazards Regression analysis[8, 11, 13, 15–20, 22, 23], usually using stepwise selection to identify candidate predictors (e.g., based on a p-value; forwards or backwards), with one model using the Bayesian Information Criterion[19]). One model, the Australian National University Alzheimer’s Disease Risk Score (ANU-ADRI), was developed using an Evidence-Based Medicine Approach rather than through a data analytical approach[4] and another, the Brief Dementia Screening Indicator (BDSI) was developed using data synthesis based on the best dementia predictors identified in four different cohort studies[20]. When computed, simple risk scores have been derived from the model’s Beta-[4, 13, 16, 19, 20] or logit-coefficients[21]. This is similar to the methodology employed for risk model development in other fields of medicine such as cardiovascular disease[27, 28]. No models have yet been developed using systems biology or neural network approaches. Neural networks simulate the functions of neurons of human brains which can interact for processing data and learning from experiences [29]. Given the potential complexity of the risk factor variables used in dementia risk prediction, neural networks may also hold promise although this has yet to be evaluated.

### Risk Models

Models can be broadly divided into the following categories: (1) demographic only models; (2) cognitive based models (incorporating cognitive test scores, with or without subjective memory/cognitive complaint indicators or demographic data); (3) health variables and health risk indices (incorporating self-reported or objectively measured health status); (4) genetic risk scores including APOE, PICALM (Phosphatidylinositol binding clathrin assembly protein), CLU (Clusterin) and other genes associated with AD (i.e., BIN1, CR1, ABCA7, MS4A6A, MS4A4E, CD2AP, EPHA1 and CD33), either alone or in combination with non-genetic variables; and, (5) multi-variable models typically incorporating demographic, health and lifestyle measures. Table 3 shows comparisons of the model components between this and the 2010 review and illustrates that there is large variability in model components and differences across the two reviews.

**Table 3:** Component Variables Used (Either Alone or in Combination) in the Different Risk Prediction Models (Previous and Current Review).

**Demographics**	Age	Age
	Education	Education
	Sex	Sex
		Race/Ethnicity
**Subjective Cognitive Complaints/Impairment**	Difficulty remembering recent events &/or difficulty remembering short list of items (Informant reported)	Self-reported difficulty with short-term memory (“Do you frequently have difficulties in retaining or remembering new simple information?”)
	Self-reported trouble “keeping mind on things” >3 days/week	Self-reported “trouble keeping mind on things” >3 days/week
	Subjective memory complaint	Subjective memory impairment (‘‘Do you feel like your memory is becoming worse?”—with and without worry)
**Functioning**	Time to put on and button a shirt	IADL scale
		IADL score (Four items: using the telephone, managing treatment, handling finances, and using transportation)
		IALD item “Does your patient need help from others to manage money or medications?”
**Neuropsychological Tests**	Activity Recall (recall of all tests worked on during the interview)	ADAS-Cog Battery (Word Recall, Command, Construction, Naming, Ideation Praxis, Orientation, Word Recognition, Remembering Instructions, Spoken Difficulties, Word Finding, Comprehension, Concentration)
	ADAS-Cog (word not recalled)	Benton Visual Retention Test (Form F MQ)
	Block Design (Wechsler Adult Intelligence Scale-Revised)	CAMCOG (Total score, Memory (Remote, Recent, Learning, composite score), Non-memory (Orientation, Language Comprehension, Language Expression, Attention & Calculation, Praxis, Abstraction, Perception, composite score))
	Boston Naming Test (subtests)	CERAD Battery (Total score, Constructional Paraxis Recall, Word List Learning, Word List Recall, Boston Naming Test)
	Buschke Selective Reminding Test	Clock Drawing Test
	CAMCOG (Total score, Memory, General Knowledge, Attention & Calculation)	Copying (intersecting pentagons)
	CASI (Total score, Semantic Memory, Episodic Memory, Attention, Concentration/Mental Manipulation, Orientation, Visual Construction, Abstraction & Judgment)	Delayed Recall (3 words)
	Clock drawing	Digit Symbol Substitution Test
	Clock setting	Free and Cued Selective Reminding Test (Free recall score, Total recall Score)
	Clock reading	Instruction (Paper folding)
	Copying (cube, coils and interlocking infinity loops)	Isaacs Set Test
	Digit Letter Test	Logical Memory I (Immediate Recall)
	Digit Symbol Substitution Test	MMSE (Total score, Episodic Memory Subtest)
	Free and cued recall of recognisable words	Rey-Auditory Verbal Learning Test (Short delayed recall score)
	Free and Cued Selective Reminding Test	Stroop Test
	Free recall of rapidly and slowly presented random words	Trail Making Test (Part A and B)
	Fuld Object Memory Evaluation (FOME) Recall Test	Vascular Dementia Assessment Scale Cognitive Battery (Delayed recall of the ADAS-Cog 10 Word Lists, Symbol Digit Test, Digit Span Backwards, A Maze Task, Digit Cancellation Task, Animal Naming)
	Identical Pictures	Verbal Fluency (Animals)
	Intra-categorical Delayed Selective Reminding Test (IDSR-7)	Wechsler Memory Scale Information Subtest
	List-generating fluency	
	Memory for Text	
	Memory Scale Information Subtest (WAIS)	
	Mini Mental State Examination (MMSE)	
	Modified MMSE (3MS: total score and individual items)	
	Paired-Associate Learning Test	
	Rey-Auditory Verbal Learning Test (Short delayed recall score)	
	Reid Memory Test	
	Rey Figure (Delay recall)	
	Screening Instrument for Cognitive Impairment & Dementia	
	Similarities (Subtests)	
	Trail Making Test (Part B)	
	Verbal Fluency (animals, fruits, flowers vegetables, groceries, letters: ‘ta’, F, A and S)	
	Vocabulary subtest of WAIS	
**Health (Objective and Self Reported)**	Angina	Acute metabolic events (severe hyperglycaemic or hypoglycaemic events)
	Aortic calcification	Anti-hypertensive agents
	Arthritis	Arthritis or rheumatism
	Atrial fibrillation	Body Mass Index
	Body Mass Index	Carotid endarterectomy
	Calf pain when walks, ceases when halts	Central obesity
	Chest pain when excited	Cerebrovascular attacks
	Chest pain when walking up hill or fast	Chest problems
	Chronic heart failure	Cholesterol
	Chronic obstructive pulmonary disease	Congestive heart failure
	Claudication	Coronary artery bypass surgery/graft
	Coronary artery bypass surgery	Cough
	Defective ventricular conduction	Dental problems
	Depression	Denture fit
	Diabetes	Depressed mood
	Dizziness when suddenly stands up	Depression
	Extrapyramidal score (including measures of tone (rigidity, cogwheeling, nuchal rigidity), bradykinesia (slowed fine finger movements, reduced arm swing, and an overall clinical assessment of the presence of bradykinesia), resting tremor, postural flexion, and the glabella tap)	Depressive symptoms (including CES-D and DSM-III-TR Classification A1: depressed mood, A2: loss of interest, A3: change of appetite, A4: sleep disturbance, A5: psychomotor change, A6: loss of energy, A7: worthlessness, A8: concentration difficulty)
	Heart disease	Diabetes
	Hypercholesterolaemia (self-reported)	Diabetic foot
	Hypertension (including hypertension currently treated)	Ear trouble
	Internal carotid artery thickness	Eye trouble
	Myocardial infarction	Eyesight trouble
	Parkinson disease	Feet or ankle trouble
	Peripheral artery disease	Fractures (any)
	Pulmonary congestion	Glycosylated haemoglobin (HbA1c)
	Second heart sound abnormal	Head trauma
	Sinus tachycardia	Hearing trouble
	Stroke	High density lipoprotein cholesterol level
	Systolic blood pressure	Hypertension
	Transient ischaemic attack	Incontinence (urinary: lose control of bladder and faecal: lose control of bowels)
	T-wave abnormalities	Kidney trouble
	Total cholesterol level	Microvascular disease (diabetic retinal or end-stagerenal)
		Myocardial infarction
		Nose stuffed up or sneezing
		Percutaneous transluminal coronary angioplasty
		Peripheral artery disease
		Poor lung function
		Skin problems
		Stomach trouble
		Stroke
		Systolic blood pressure
		Total cholesterol level
		Traumatic brain injury
		Waist-Hip-Ratio
**Lifestyle**	Alcohol	Alcohol
	Physical activity	Physical activity/Exercise
	Smoking	Smoking
**Diet**		Folic acid
		Fish intake
**Genetics**	APOE	ABCA7
		APOE
		BIN1
		CD2AP
		CD33
		CLU
		CR1
		EPHA1
		MS4A6A
		MS4A4E
		PICALM
**MRI**	Enlarged ventricles	
	White matter disease	
**Statistics/Methodology**	Reliable change indices	
	Within persons across test variability	
**Other**	Family history of dementia (Informant report)	Cognitive activity
	Walking speed (5 meter returned walk)	Clinical Dementia Rating Scale—Sum of Boxes (Clinician & algorithm based)
		International Working Group Consensus Criteria for Mild Cognitive Impairment (MCI)
		Self-reported Health (how good & report of any health problems)
		Social network and engagement

**Abbreviations: ADAS-Cog** Alzheimer's Disease Assessment Scale-cognitive; **IADL** Instrumental Activities of Daily Living; **CAMCOG** Cambridge Cognitive Examination; **CASI** The Cognitive Abilities Screening Instrument; **CERAD** Consortium to Establish a Registry for Alzheimer’s Disease; **CES-D** Center for Epidemiologic Studies **Depression Scale**; **DSM-III-R** Diagnostic & Statistical Manual of Mental Disorders–3rd Edition Revised; **IDSR-7** Intra-categorical Delayed Selective Reminding Test; **MMSE** Mini Mental State Examination; **WAIS** Wechsler Adult Intelligence Scale

Differences are mainly in the addition of novel (non-traditional) dementia risk variables[25], information on diet[4], depression symptomology[4, 13, 24], ethnicity[14, 20, 23], and extension of genetic analysis to include non-APOE genes[17]. In addition, fewer cognitive tests are used and there is a smaller pool of candidate risk factors, likely due to more evidence being available.

### Model Diagnostics

Performance of models has been assessed using measures of discriminative accuracy (e.g., AUC/c-statistic), sensitivity, specificity, positive predictive value (PPV), negative predictive value (NPV), internal calibration, LR+/LR-, the net reclassification index (NRI) and the integrated discrimination improvement (IDI) statistic. Discriminative accuracy was measured in all studies and ranged from low 0.49[4] to moderate 0.89[9]. Cut-off points with sensitivity and specificity estimates were only reported in five studies[7, 19, 22, 23, 26]. Where available, cut-off points were determined as follows: (1) maximisation of Youden’s index (Formula = Sensitivity + Specificity– 1)[22, 23, 26]; (2) computed by cross validation to correct for optimism as a result of validation on the learning data to correspond to a sensitivity value[7]; or, (3) defining the cut-off scores as those values with high specificity and increased PPV[19]. No model reported a cut-off score with both sensitivity and specificity over 80%. Three studies reported PPVs: (1) 9 to 41% (range across different length of follow-up interval and educational level)[7]; (2) 6.6 to 49.9% (range across different cut-off scores)[23]; and, (3) 14.7% with a cut-off that provided sensitivity of at least 80%[19]. Two studies reported NPVs: range across different cut-off scores: 86.0 to 99.0%[7] and 97.7%[19]. NPV should be higher than the proportion of subjects who did not have the outcome of dementia (i.e., the “stable” subjects), if the prediction is better than chance. Since the proportion of subjects not becoming demented was generally >85% (and always >70%)–as inferred from Table 2 –a high NPV is expected, because the number depends on the prevalence of the disease in the sample. The same logic can be applied to PPV. It should be noted that because the proportion of subjects becoming demented (or not) varies among the populations, variations in PPV and NPV do not necessarily reflect differences in performance of the models. Only one study[26] reported LR+/LR- and found that a neuropsychological prediction model provided a clinically important change in pre- to post-test probability of converting to dementia (all cause) over 5 and 10 years follow-up.

Model calibration was rarely reported, and where reported indicated good fit[13, 14, 20]. Reclassification indices including NRI and the IDI statistics that test the addition of variables to risk models, were used in two studies[14, 15]. The first study evaluated the influence of the APOE genotype on the accuracy of AD risk assessment using NRI and found a significant improvement (NRI 0.18, Z_NRI_ = 2.47, P = 0.01) compared to a non-APOE model; the IDI was estimated as 6.25 (Z_IDI_ = 3.75, P <0.001)[15]. The second used NRI and IDI to assess the improvements in performance of the Cardiovascular Risk Factors, Aging and Dementia (CAIDE) risk score by adding new risk factors[14]. Here, both the NRI and IDI showed no model improvements with the additional variables.

### Models for Specific Subgroups of Individuals

One study developed a risk score, the Type-2 Diabetes Specific Dementia Risk Score (DSDRS), for predicting 10-year incident dementia, in a primary care setting, in a large cohort of individuals (N = 29,961) with type II diabetes[13]. The model incorporated age, education, microvascular disease, diabetic foot, cerebrovascular disease, cardiovascular disease, acute metabolic event and depression and was well calibrated and externally validated with moderate levels of predictive accuracy (AUC = 0.74 development cohort vs. AUC = 0.75 validation cohort). In another study, model development was undertaken in a sample stratified by education level (low: no elementary school diploma vs. high: secondary school or university) and follow-up time (3 vs. 10 years)[7]. This resulted in four different models that varied by education and length of follow-up as shown in Table 2.

### Model Validation

**Fig 2:**
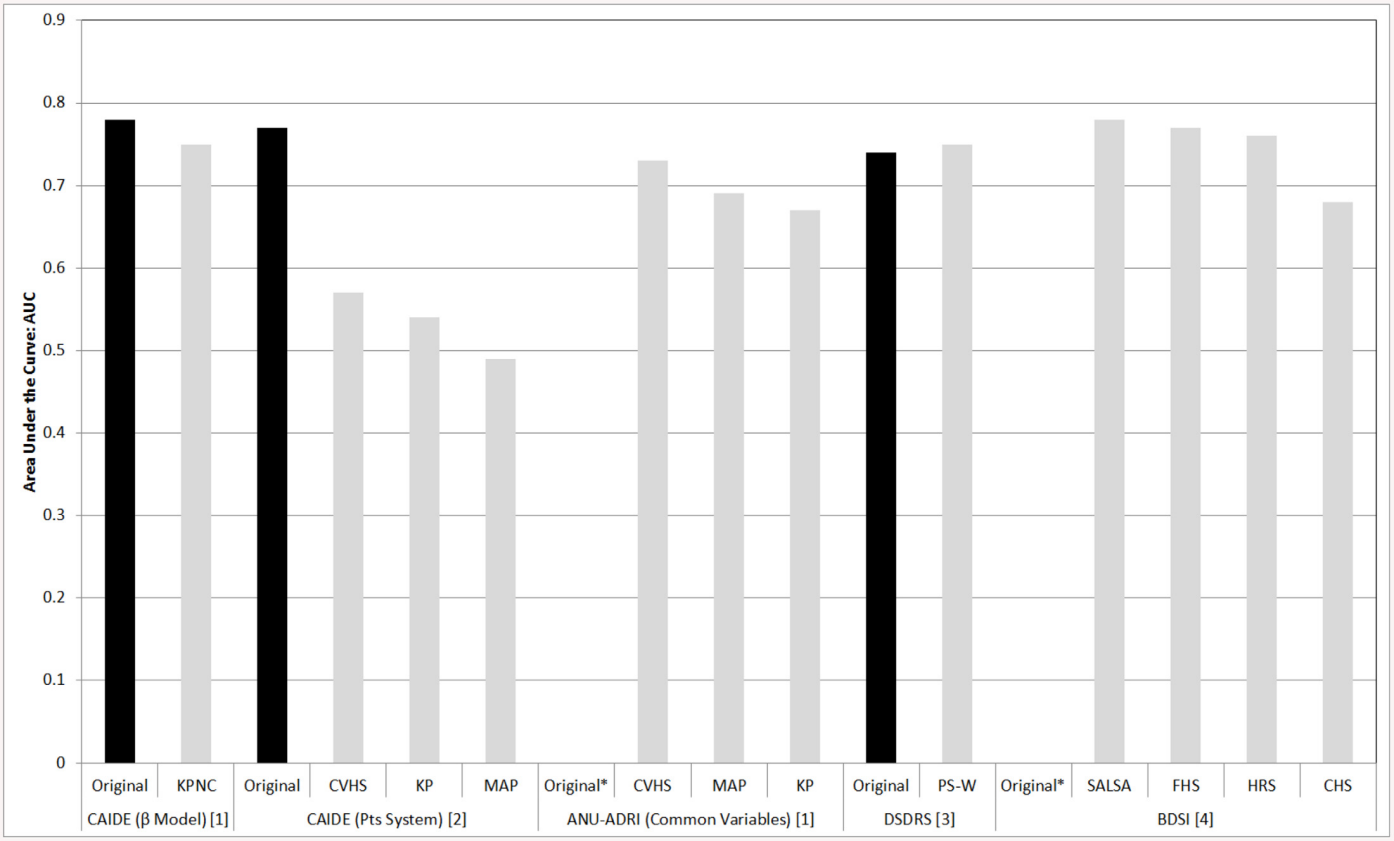
Comparison of AUC indices in development vs. validation cohorts across different dementia risk prediction models. **Key:** BDSI, Brief Dementia Screening Index; CAIDE, Cardiovascualr Risk Factors, Aging and Dementia; CHS, Cardiovascular Health Study; CVHS, Cardiovascular Health Cognition Study; DSDRS, Type-2 Diabetes Specific Dementia Risk Score; FHS, Framingham Heart Study; KP, Kungsholmen Project; KPNC, Kaiser Permanente Medical Care Program of Northern California; MAP, Rush Memory and Aging Project; PS-W, Pathways study cohort from Washington; Pts, Points; SALSA Sacramento Area Latino Study on Aging. **References** [1] Anstey KJ, Cherbuin N, Herath PM, Qiu C, Kuller LH, Lopez OL, et al. A Self-Report Risk Index to Predict Occurrence of Dementia in Three Independent Cohorts of Older Adults: The ANU-ADRI. PLoS One. 2014;9(1):e86141; [2] Exalto LG QC, Barnes D, Kivipelto M, Biessels GJ, Whitmer RA. Midlife risk score for the prediction of dementia four decades later. Alzheimers Dementia. 2013; [3] Exalto LG, Biessels GJ, Karter AJ, Huang ES, Katon WJ, Minkoff JR, et al. Risk score for prediction of 10 year dementia risk in individuals with type 2 diabetes: a cohort study. The Lancet Diabetes and Endocrinology. 2013; [4] Barnes DE, Beiser AS, Lee A, Langa KM, Koyama A, Preis SR, et al. Development and validation of a brief dementia screening indicator for primary care. Alzheimers Dementia. 2014:S1552-5260. **Notes** * No development dataset. Rather, model tested in different cohorts.

Only four studies have undertaken validation[4, 13, 14, 20]. Differences between the AUCs in the development and validation cohorts for the different models tested are shown in Fig 2.

#### CAIDE

The CAIDE model was developed in sample of participants from Finland (N = 1,409, age range: 39 to 64 years) and uses risk factors in midlife to estimate an individuals’ risk of later life dementia (mean follow-up time = 20 years). The model incorporates age, education, sex, cholesterol level, BMI and systolic blood pressure (with and without APOE e4 status)[30]. Using data from the Kaiser Permanente (n = 9,480; age range: 40 to 55 years; mean follow up-time = 36.1 years) a similar AUC to the original study was reported (0.75 validation vs. 0.78 original cohort)[14]. Furthermore, when the Kaiser Permanente sample was stratified by ethnicity the CAIDE model was found to predict dementia well across different ethnicities including Asian (AUC = 0.81), Black (AUC = 0.75) and White (AUC = 0.74). These estimates are comparable to those reported in the original publication (model development dataset AUC = 0.78)[30]. This study also attempted to improve the discriminative accuracy of the CAIDE score with the addition of new variables such as central obesity, depressed mood, diabetes, head trauma, poor lung function and smoking but no significant improvement was shown[14].

When using the CAIDE risk score for predicting dementia in three older aged cohorts, the Rush Memory and Aging Project, the Kungsholmen Project and the Cardiovascular Health and Cognition Study, validation was found to be poor (AUC range all-cause dementia: 0.49 to 0.57; AUC range AD: 0.49 to 0.57)[4]. Interestingly, excluding BMI or BMI and cholesterol level together, modestly increased discriminative accuracy for all-cause dementia (AUC range: 0.55 to 0.60) and AD (AUC range 0.55 to 0.58)[4]. This result suggests that these variables may not be as important for predicting dementia in later vs. midlife. Indeed, in some studies of older aged cohorts higher BMI, cholesterol levels and blood pressure are found to be protective against dementia[31]. Therefore, poor transportability of the CAIDE model to these three cohorts may be due to the fact that the development dataset was a midlife cohort and the validation datasets were from older aged cohorts (Mean at baseline range: 72.3 to 81.5 years). The results could also be due to attrition rates as only 3% were lost to follow-up in the original study[30] compared to more than 20% of participants being lost in the three validation cohorts[4].

#### ANU-ADRI

Using an evidence synthesis approach to model development, the ANU-ADRI model was developed to assess a persons’ risk for later life AD (i.e., over 60 years of age) based on exposure to 11 risk and four protective factors including: age, education, sex, BMI, diabetes, depression, cholesterol, traumatic brain injury, smoking, alcohol use, physical activity, pesticide exposure, social engagement, cognitive activity, and fish intake. Validation of the ANU-ADRI[4] score produced moderate levels of discrimination for dementia when between eight to 10 of the different risk/protective variables were mapped across three studies (AUC range: 0.64 to 0.74). The studies included the Rush Memory and Aging Study (N = 903), the Kungsholmen Project (N = 905) and the Cardiovascular Health Cognition Study (N = 2,496). The variables mapped included: demographic (age, gender, education), health (diabetes, traumatic brain injury, depressive symptoms), cognition (cognitive activity) and lifestyle factors (social network and engagement, smoking, alcohol, physical activity). When only common variables from all cohorts were used (n = 6 variables including: age, sex, education, diabetes, smoking, alcohol) the AUCs were: 0.69 (95% CI 0.65 to 0.73), 0.68 (0.63 to 0.70) and 0.73 (0.71 to 0.76) in the Rush Memory and Aging Study, the Kungsholmen Project and the Cardiovascular Health Cognition Study, respectively. The authors did not test whether the differences in AUCs when common variables were mapped across the different cohorts were statistically significant. These results are interesting and raise the question as to whether all or just some risk factors are needed to accurately predict future disease. It should be noted that one explanation for differences in AUC estimates could be variation in age. In particular the Kungsholmen Project, which included participants born before 1912 (baseline age > 75), was older than the other two samples (Rush Memory and Aging Study baseline age > 53, Cardiovascular Health Cognition Study > 65). The study also investigated the effect of gender on discriminative accuracy of the ANU-ADRI within each cohort and found only slight differences: the reported 95%CIs for males and females overlapped suggesting that any differences in discriminative accuracy were not significant.

#### BDSI

The BDSI was developed with a three-step approach in four cohort studies including: the Cardiovascular Health Study, Framingham Heart Study, Health and Retirement Study, and the Sacramento Area Latino Study on Aging[20]. First, a list of potential predictive factors available in most or all cohorts was identified. Second, in each cohort, variables most predictive of dementia at six years were identified independently. Third, a subset of variables that were consistently found in all four cohorts was identified and used in the model including demographics (age, education), health (history of stroke, diabetes mellitus, BMI, depressive symptoms) and lifestyle (assistance needed with money or medications) factors. The c-statistic for predicting 6-year incident dementia varied between the 4 cohorts from 0.68 to 0.78. Sensitivity analyses, using data from the Health and Retirement Study and Cardiovascular Health Study, suggested that discrimination was good across different race/ethnic groups: Health and Retirement Study (c-statistic = 0.75 Whites, 0.70 Blacks, 0.71 Latinos) and the Cardiovascular Health Study (c-statistic = 0.70 Whites, 0.65 Blacks)[20].

### Cost Considerations

One study considered the impact on discriminative accuracy of modifying the calculation of a resource intensive risk score to incorporate less expensive measures. The original resource intensive model, the Late Life Dementia Risk Index, included demographic (age), lifestyle (alcohol consumption), neuropsychological (Modified Mini Mental State Examination (MMSE) score and Digit Symbol Substitution Score), medical (history of coronary bypass surgery and BMI), physical functioning (time to put on and button a shirt in seconds), genetic (APOE), cerebral magnetic resonance imaging (MRI) (white matter disease and enlarged ventricles), and carotid artery ultrasound (internal carotid artery thickness >2.2mm) measures, and had good discrimination for the prediction of 6-year incident dementia (c-statistic = 0.81, 95%CI: 0.79 to 0.83)[32]. The revised model, the Brief Dementia Risk Index, incorporated age, neuropsychological testing (3 word delayed recall, interlocking pentagon copying, verbal instructions (paper taking and folding), four legged animal naming task (30 seconds)), self-reported attention difficulties (3 or more days per week in the last month), medical history (stroke, peripheral artery disease, or coronary artery bypass surgery and BMI) and alcohol consumption and had a significantly lower discriminative accuracy (c-statistic = 0.77, p<0.001), but was able to categorize subjects as having low, moderate, or high risk of dementia with similar accuracy compared to the more resource intensive score[21].

## Discussion

The results from the review show that many new models for dementia risk prediction have been developed over the last five years. There have also been significant changes to the types of variables used when compared to the previous review. However few studies have addressed the issues of external validation and cost in using the risk model.

### Strengths and Limitations

The strengths of this review are its systematic approach and inclusivity. There are some limitations. It is difficult to synthesise the literature on dementia risk prediction due to the large variability across studies in follow-up length (range: 1.5 years[9] to 17 years[15]), sample age (range: 40 to 99 years), outcomes tested (e.g., AD vs. all-cause dementia vs. dementia subtypes, quality of the diagnosis), source of population (volunteer vs. population representative) and the different variables incorporated into the prediction models. As such a meta-analysis was not possible. Furthermore, any meaningful conclusions for population screening are limited by the lack of cost-effective analysis and limited assessment of model transportability.

### Clinical Implications

There is currently a clinical drive towards timelier diagnosis particularly in developed countries such as the UK with the introduction of primary care direct enhanced services looking to identify those at risk of developing dementia e.g. stroke, diabetes, cardiovascular disease[33]. A risk prediction tool, particularly in at risk populations such as diabetes[13] could further enhance existing services. However, if model development in the field of dementia continues at its current pace and if dementia risk prediction is found to be useful and cost-effective, then researchers and possibly clinicians will face difficult choices regarding which model to apply, particularly as study comparison is difficult.

#### Variables in the Prediction Models and Comparison to Results from the First Review

Compared to the earlier review [1] elements common to the majority of risk scores include age, education, measures of cognition and health. However, new developments in dementia risk prediction include non-APOE genes and genetic risk scores[17, 18], testing of non-traditional dementia risk factors[25], incorporation of information on diet[4], physical function[4], physical activity/exercise and ethnicity[16] into risk modelling, and model development in specific subgroups of the population (e.g., individuals with diabetes[13] and those with low vs. high educational attainment[7]) and over different follow-up times. Furthermore, fewer cognitive tests have been used in prediction models, reflecting our increasing knowledge of risk and protective factors. Despite the dramatic increase in the number of models and novel risk scores, discriminative accuracy has not changed to a significant degree when compared to the previous review (range in the 2010 review: 0.49 to 0.91 vs. range in this review: 0.49 to 0.89). Aside from cognitive based models, generally, the best models are those that incorporate multiple risk factors across different variable categories (e.g., demographic, cognition, physical and health). Within the limits of our relatively limited knowledge of the genetic factors that influence dementia risk, where statistically tested, addition of novel (non-APOE) risk factors to prediction models do not appear to significantly increase discriminative accuracy[17]. In contrast, the APOE genotype, at least in some studies, appears to be informative. Future work into polygenic risk scores would help.

Further there has been a drive towards more accessible and potentially modifiable variables. Modifiable variables are important as they have the potential to be specifically targeted in primary or secondary prevention. There is now also evidence that around a third of AD cases worldwide may be due to modifiable risk factors [34]. With risk models likely to be used in the primary care setting, the availability of imaging variables may be difficult to obtain nor do they significantly improve discrimination in prediction of dementia beyond the more readily published multifactorial models [35].

### Stratified Analyses

Results from stratified analysis suggest that unique dementia risk prediction models may need to be developed depending on follow-up length (e.g., BMI and hypertension may be more important in mid-life compared to later life models)[7, 23, 26, 36–38], an individual’s education level (found in one study and requires replication) [7], health status (e.g., diabetes)[13], APOE status[23] and the outcome tested (e.g., most studies focus on all-cause dementia and different models maybe needed depending on dementia subtype)[19]. Further research is required to develop risk models in samples stratified by other confounding factors known to influence the timing and presentation of dementia symptoms (such as mid vs. later life) as well as investigate the interactions between different risk variables (e.g., such as AOPE status and age).

### Model Transportability

Before a risk prediction tool can be used in clinical practice or for research, transportability of the model outside the cohort from which it was developed needs to be assessed. Only four studies have externally validated dementia risk prediction models and the results were mixed[4, 13, 14, 20]. The DSDRS model was developed and validated with moderate but similar levels of discrimination (AUC 0.74 development vs. 0.75 validation)[13]. This is in comparison to the CAIDE score (originally developed for midlife), which was poorly validated in three separate (older) cohorts (AUC 0.77 development vs. AUC range 0.49–0.57 validation)[4]. However, it is important to note that in this validation of the CAIDE score rather different populations were used, that varied by age (mid vs. later life). Indeed, factors like higher BMI, blood pressure and cholesterol levels are associated with lower incidence of dementia in the oldest old [31]. In contrast, the CAIDE score was found to transport well when the test population better resembled the original population (i.e., was a mid-life cohort)[14]. Generally, external validation is difficult largely due to the lack of available cohorts with which to test models in terms of follow-up times, data collected, age groups and risk variable measurement.

Most studies have been developed in datasets from North America (N = 12 studies), with others developed in the UK (N = 1), Japan (N = 1), Austria (N = 1), the Netherlands (N = 2), Pan-Europe (N = 1), Germany (N = 3) and France (N = 1). Whether the different models are applicable across countries that vary by health and wealth is not known. Further, no models have been developed for predicting other dementia sub-types, such as vascular dementia or dementia with Lewy Bodies or for predicting different disease severity (e.g., mild, moderate and severe dementia). This may have implications for treatment options.

### Issues Around Cost

The ability to assess the relative cost of calculating the different risk models and compare this against the model’s accuracy will significantly influence recommendations about possible protocols for screening for dementia risk. Further, the incorporation of readily accessible primary care related factors would be most useful for application within clinical and population based settings. Three studies [4, 20, 21] have considered time and/or financial implications in risk score calculation. However, in one study it was found that reducing the cost of risk score computation by removing the need for MRI, ECG and detailed neuropsychological measures and replacing them with less resource intensive variables (e.g., more detailed self-reported health history and simple cognitive test items) resulted in a significant decline in discriminative accuracy[21, 32]. Thus raising the issue of what is the best information and minimum data set needed for accurate dementia risk prediction. It is important to note that current models have not yet utilized cerebrospinal fluid or positron emission tomography data as recommended for classifying AD and its preclinical stages in new (clinical and research) criteria for AD and its preclinical/prodromal states[39, 40]. Although these factors can be used to assist dementia diagnosis, the feasibility and acceptance of incorporating them into risk prediction models and, particularly in population-based settings, would likely be low.

## Conclusions

Before a risk assessment tool can be implemented we need to know its discriminative accuracy, predictive value, cost-effectiveness, transportability (e.g., to particular populations, ages and gender etc.), and the general availability of its variables (e.g., to enable cross-study comparison and result verification). We must also consider the design implications of the model: Are we interested in highly sensitive indicators of near term (i.e., 3-years) incidence of dementia? What operating characteristics are optimal for longer-term (5–10 years) predictive models? Are we contemplating a stratified approach, whereby low-cost screening identifies subjects with higher risk for more detailed and costly assessments?

It is not possible to state with certainty whether there exists one model that can be recommended for dementia risk prediction in population-based settings. This is largely due to the lack of risk score validation studies. Consideration of the optimal features of new models should largely focus on methodology (model development and testing) and the cost and acceptability of deriving the risk factors. Further work is required to validate existing models or develop new ones, as well as to assess their cost-effectiveness and ethical implications, before applying the particular models in population-based or clinical settings. While it is difficult to make a recommendation regarding which model, we nonetheless offer some recommendations of the optimal features for new models (see Table 4).

**Table 4:** Optimum features of study design and variables selected for dementia risk prediction models.

***Data & Data Analysis***
Minimal attrition or use of methodology that accounts for attrition (e.g., loss of follow-up and death)
Considerations of the description of population, diagnostic method and follow-up time
Examine internal validity (i.e., equivalent performance in different subgroups)
External validation
High AUC/c-statistic (closer to 1 the better)
Consideration of whether to prioritise sensitivity or specificity and the implications of doing so
***Data Presentation***
Sensitivity and specificity given at multiple cut-off points
Confidence Intervals of each statistic and if comparisons are made, use of a formal method of statistical inference
***Risk Factor Timing***
Special attention to mid-life risk factor ascertainment in older subjects (e.g., risk factors for dementia in mid-life may not be associated with dementia in older subjects)
***Patient Acceptability***
Risk variable attainment (e.g., cost and ease of acquisition of the data from a patient as well as health care provider point of view)
Risk score calculation (acceptability to the patient/health care provider)
***Ease of Access to Variables***
Cost
Invasiveness

## Supporting Information

S1 PRISMA Checklist PRISMA 2009 Checklist. (DOC)
